# Biomechanical stability of coronoid fractures with different fragment heights in the terrible triad of the elbow: CT measurement and finite element analysis

**DOI:** 10.3389/fbioe.2026.1864231

**Published:** 2026-07-21

**Authors:** Jingrui Zhao, Yuandong Liu, Qiang Xu, Chen Niu, Bo Yu

**Affiliations:** 1 Shandong University of Traditional Chinese Medicine, The First Clinical Medical College, Jinan, Shandong, China; 2 Shandong University of Traditional Chinese Medicine Affiliated Hospital, Department of Orthopaedics, Jinan, Shandong, China

**Keywords:** finite element analysis, Kirschner wire, process fracture heigh, the coronoid process of the ulna, the terrible triad of the elbow

## Abstract

**Background:**

Coronoid fracture height is an independent factor influencing elbow joint stability. In the terrible triad of the elbow, coronoid fractures are typically manifest as small transverse fractures involving the coronoid tip. Anatomical reduction and stable fixation of the coronoid fragment are therefore critical for restoring concentric reduction and reliable stability of the elbow joint. This study investigated the effect of coronoid fracture fragment height on elbow joint stability under dual parallel Kirschner wire fixation and explored the quantitative relationship among fragment height, stress distribution, and displacement. The findings aim to facilitate a transition in elbow injury management from experience-based treatment toward data-driven precision therapy.

**Methods:**

Based on computed tomography data, the coronoid fracture fragment height was measured in 60 patients with the terrible triad of the elbow. Four finite element models representing coronoid fracture heights of 15%, 30%, 45%, and 60% were established. A finite element analysis model was constructed to simulate coronoid fracture fixation using dual parallel Kirschner wires. Pull-out, horizontal, and rotational displacement simulations were performed for each model. Stress distribution within the fixation devices and fracture fragments, as well as fragment displacement, were recorded. Von Mises stress distributions were generated to evaluate the mechanical behavior and stabilizing effect of the fixation construct.

**Results:**

Among the four models, the 30% fracture height model demonstrated superior pull-out resistance, followed by the 45% fracture height model. In the horizontal displacement simulation, the 30% fracture height model demonstrated stronger resistance to horizontal displacement, whereas the 45% fracture height model exhibited more favorable stress distribution characteristics. In the rotational displacement simulation, fracture fragment displacement increased progressively with increasing fracture height; however, the displacement in all models remained below 1.00 mm.

**Conclusion:**

Under the study conditions, dual parallel K-wire fixation provided effective stabilization when the fracture fragment height ranges from 30% to 45% of the total coronoid height, demonstrating relatively favorable biomechanical stability. This fixation strategy represent a relatively stable surgical option for the management of coronoid fractures associated with the terrible triad of the elbow and provide a biomechanical basis for postoperative rehabilitation and early mobilization. However, clinical decisions should be made in conjunction with the assessment of associated ligamentous injuries, radial head fractures, and overall elbow joint stability.

## 1 Introduction

Coronoid fractures associated with the terrible triad of the elbow are typically manifest as small transverse fractures involving the coronoid tip (Morrey and An, 2005). As a fundamental osseous stabilizer of the ulnohumeral joint, the ulnar coronoid process is essential for maintaining anteroposterior and rotational stability, while also providing resistance to varus and valgus stresses (Al-Ani et al., 2022). Surgical reduction and fixation of the coronoid fragment are therefore critical for restoring concentric reduction and reliable stability of the elbow joint (Wang et al., 2023). In this study, three-dimensional (3D) computed tomography (CT) was used to measure the height of coronoid fracture fragments in the terrible triad of the elbow. Fixation using two parallel Kirschner wires (K-wires) inserted in a posterior-to-anterior fashion, offers several clinical advantages, including technical simplicity, ease of hardware removal, and low cost (Kumar et al., 2020). Current studies mainly focus on the selection of surgical approaches, improvements in K-wire fixation techniques, and postoperative functional rehabilitation, whereas systematic investigations into the biomechanical relationship between fracture fragment morphology and fixation methods remain limited (Fahs et al., 2024). In this study, finite element analysis (FEA) was employed to investigate the biomechanical characteristics of dual parallel K-wire fixation in coronoid fractures with different fragment heights. The effect of fragment height on fixation stability was systematically evaluated, revealing a quantitative relationship among fragment height, stress distribution, and displacement. Through sensitivity analysis, this study aims to promote a transition in elbow injury management from experience-based treatment to data-driven precision therapy. The findings may provide quantitative biomechanical evidence to refine surgical indications and optimize treatment strategies for coronoid fractures of different fragment heights.

## 2 Materials and methods

### 2.1 Measurement analysis and feature extraction of CT data

Cases clinically diagnosed with the “terrible triad of the elbow” were collected at Shandong University of Traditional Chinese Medicine Affiliated Hospital from 1 March 2022, to 1 March 2025. A total of 60 CT scans of elbows were included, comprising 30 males and 30 females aged between 18 and 60 years. The CT scans were acquired with a slice thickness ranging from 0.5 to 1.0 mm and were stored in Digital Imaging and Communications in Medicine (DICOM) format. Inclusion criteria: (1) Patients clinically diagnosed with the terrible triad of the elbow; (2) Patients with normal musculoskeletal development and anthropometric characteristics, whose height, weight, and body mass index (BMI) fall within normal ranges; (3) Patients without a history of elbow pathology or prior elbow surgery. Exclusion criteria: (1) History of upper-extremity trauma resulting in fractures, malunion, joint dislocations, or other post-traumatic deformities; (2) Congential skeletal abnormalities, including radioulnar synostosis, achondroplasia, rickets, and gigantism; (3) Bone disorders such as osteophyte formation, osteoporosis, degenerative joint disease, and osteoarthritis; and (4) Pathological fractures.

The height of the coronoid process of the ulna was defined as the vertical distance from the tip of the coronoid process to the deepest point of the ulnar trochlear notch (Sircar et al., 2023). The total height of the coronoid process was calculated as the sum of the distance from the deepest point of the trochlear notch to the fracture and the height of the coronoid fracture fragment (Doornberg et al., 2006). To evaluate the reliability of the measurement technique, 2 observers independently performed all measurements and interobserver reliability was calculated. To assess intraobserver reliability, each observer repeated the measurements after a 1-week interval. Inter-observer reliability was assessed using the intraclass correlation coefficient (ICC) based on a two-way random-effects model for absolute agreement (Anvari et al., 2018). The reliability was excellent, with an ICC of 0.992 (95% CI:0.988-0.993)for the total height of the coronoid process and 0.999 (95% CI:0.999-1.000)for the height of the coronoid fracture fragment. The mean total height of the coronoid process was 17.92 ± 1.89 mm for Observer A and 18.17 ± 1.90 mm for Observer B. Meanwhile, the mean fracture fragment heights were 6.54 ± 2.11 mm and 6.60 ± 2.12 mm, respectively. The mean fracture fragment heights was approximately 36% of the total coronoid height. According to the Regan–Morrey classification and clinically relevant thresholds (Regan and Morrey, 1989; Fahs et al., 2024), fracture fragment heights of 15%, 30%, 45%, and 60% of the total coronoid height were selected to construct representative models for subsequent finite element analysis.

### 2.2 Finite element model establishment

A healthy 28-year-old male (height: 179 cm; weight: 75 kg) with a normally developed right elbow joint was recruited for this study. The selection criteria included a normally developed elbow joint, as well as the absence of prior elbow trauma, pathological fractures, tumors, infections, skeletal deformities, metabolic bone diseases, or any other conditions that might affect the anatomy and biomechanical properties of the elbow. The elbow joint was scanned with a 320-slice spiral CT (Siemens, Munich, Germany), and the data were saved in DICOM format. These datasets were subsequently imported into Mimics 21.0 software (Materialise, Leuven, Belgium) and the 3D model of the coronoid process was reconstructed from the CT images. The model was processed in Geomagic Studio 2013 (Raindrop Geomagic Inc., USA) for denoising, smoothing, and fitting the surface. The 3D smooth solid model was developed and imported into Unigraphics NX 10.0 (Siemens PLM Software, Co., Ltd., USA) for model development. A transverse horizontal osteotomy was performed at the predetermined height of the coronoid process, and the fracture gap was set at 0.2 mm, generating four coronoid fracture models with fragment heights of 15%, 30%, 45%, and 60% of the total coronoid height. Based on considerations of fracture-height configurations, clinical practice, and biomechanical stability, stainless-steel K-wires with a diameter of 2.0 mm was selected for all models. To simulate postoperative fixation, the K-wires were inserted perpendicular to the fracture plane, traversing the coronoid fragment and extending into the proximal ulna. Finite element analysis was subsequently performed in Abaqus 2024 (Dassault Systèmes, Vélizy-Villacoublay, France), with a friction coefficient of 0.24 assigned to the fracture interface. The material properties of the internal fixation, cortical bone, and cancellous bone were defined according to the parameters listed in Table 1.

**TABLE 1:** Material data.

Material	Elastic modulus (MPa)	Poisson’s ratio	Yield strength (MPa)
Cortical bone	12,000	0.3	84
Cancellous bone	325	0.29	13
K-wire	210,000	0.3	380

**FIGURE 1:**
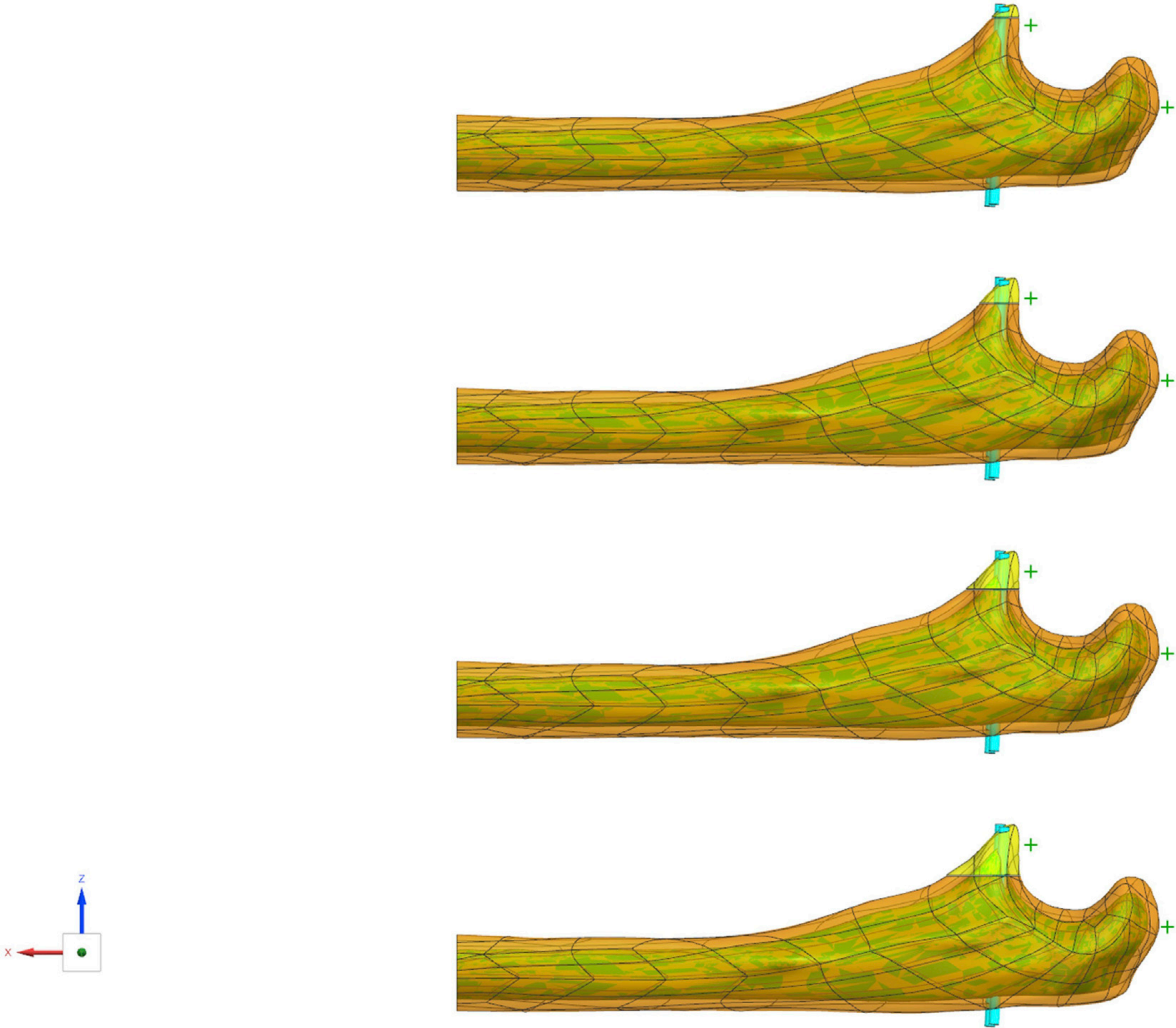
Dual parallel Kirschner wire fixation models of fractures of the ulnar coronoid process.

Pull-out, horizontal, and rotational displacement simulations were performed for each model (Figure 1). In the pull-out simulation, the coronoid fracture fragment was fixed, and a load was applied along the dorsal direction of the ulna parallel to the axis of the Kirschner wires. The load ranged from 0 to 300 N and was applied in 10 incremental steps. In the horizontal displacement simulation, the olecranon was fixed, and a load was applied at the midpoint of the articular surface of the coronoid fragment in a direction perpendicular to the articular surface toward the distal ulna. The load ranged from 0 to 300 N and was applied in 10 incremental steps. In the rotational displacement simulation, the olecranon was fixed, and a load was applied at the sublime tubercle parallel to the ulnar shaft toward the proximal ulna. The load ranged from 0 to 300 N and was applied in 10 incremental steps. The displacement and stress changes of the fracture fragments were recorded for each model, and von Mises stress distributions were obtained.

## 3 Results

**FIGURE 2:**
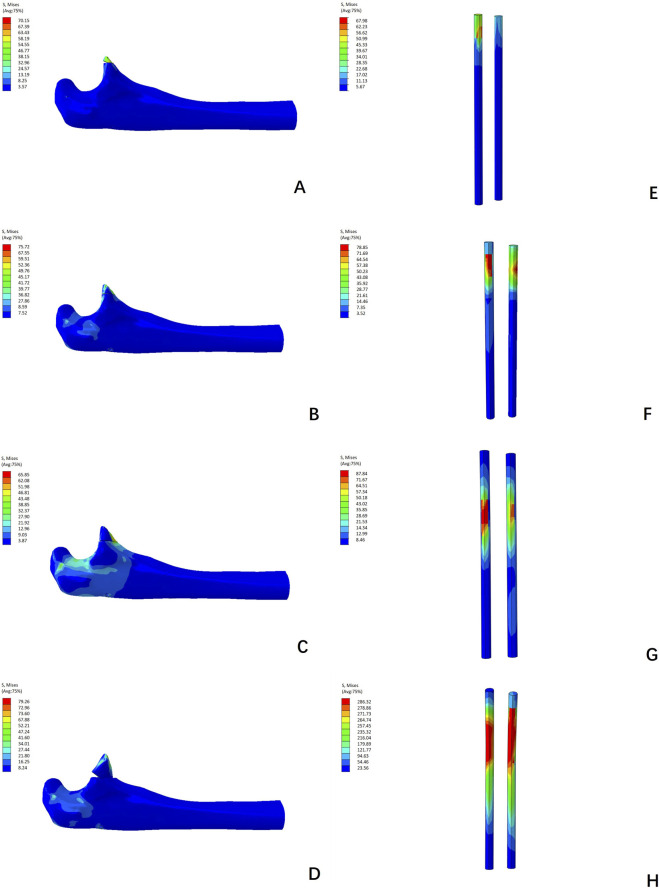
Von Mises stress distributions of the fracture fragments and K-wires in the pull-out simulation: **(A)** Von Mises stress distribution of the fracture fragment for the 15% fracture height model. **(B)** Von Mises stress distribution of the fracture fragment for the 30% fracture height model. **(C)** Von Mises stress distribution of the fracture fragment for the 45% fracture height model. **(D)** Von Mises stress distribution of the fracture fragment for the 60% fracture height model. **(E)** Von Mises stress distribution of K-wires for the 15% fracture height model. **(F)** Von Mises stress distribution of K-wires for the 30% fracture height model. **(G)** Von Mises stress distribution of K-wires for the 45% fracture height model. **(H)** Von Mises stress distribution of K-wires for the 60% fracture height model.

The displacement of the coronoid fracture fragments, as well as the stresses experienced by the fracture fragments and K- wires in each model, are presented in Table 2. In the pull-out simulation (Figure 2), the 15% fracture height model exhibited relatively large displacement of the fracture fragment. A displacement of 2.00 mm occurred at a load of 169 N. The maximum von Mises stress in the fracture fragment was 70.15 MPa, while the peak stress on the K-wires was 67.98 MPa. In the 30% fracture height model, the maximum displacement of the fracture fragment was 0.15 mm. The maximum stress in the fracture fragment was 65.72 MPa, while the peak stress on the K-wires reached 78.85 MPa. In the 45% fracture height model, the maximum displacement of the fracture fragment was 0.21 mm, with a maximum stress of 65.85 MPa in the fracture fragment and a peak stress of 87.84 MPa on the K-wires. In the 60% fracture height model, the maximum fracture fragment displacement reached 1.25 mm. The maximum stress in the fracture fragment was 79.26 MPa, while the peak stress on the K-wires reached 286.32 MPa.

**TABLE 2:** The displacement of the coronoid fracture fragments, as well as the stresses experienced by the fracture fragments and K- wires in each model. For the 15% fracture height model, the reported data were recorded at the failure threshold load (169 N). In contrast, the data for all other fracture height models were obtained under an applied load of 300 N.

Model	Max displacement (mm)	The pull-out simulation	Max K- wires stress (MPa)	Max displacement (mm)	The horizontal displacement simulation	Max K- wires stress (MPa)	Max displacement (mm)	The rotational displacement simulation	Max K- wires stress (MPa)
		Max fracture fragment stress (MPa)			Max fracture fragment stress (MPa)			Max fracture fragment stress (MPa)	
The 15% fracture height model	2.00	70.15	67.98	0.47	73.95	110.85	0.12	59.35	72.55
The 30% fracture height model	0.15	65.72	78.85	0.16	77.25	148.65	0.11	56.51	88.17
The 45% fracture height model	0.21	65.85	87.84	0.35	65.21	223.73	0.15	58.62	137.22
The 60% fracture height model	1.25	79.26	286.32	1.72	73.95	397.34	0.18	60.85	269.07

**FIGURE 3:**
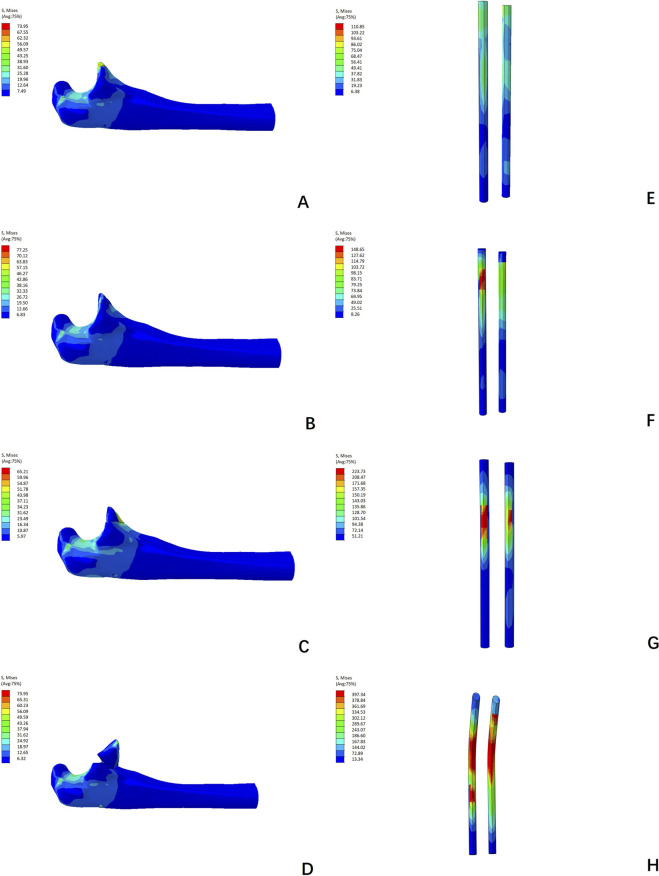
Von Mises stress distributions of the fracture fragments and K-wires in the horizontal displacement simulation: **(A)** Von Mises stress distribution of the fracture fragment for the 15% fracture height model. **(B)** Von Mises stress distribution of the fracture fragment for the 30% fracture height model. **(C)** Von Mises stress distribution of the fracture fragment for the 45% fracture height model. **(D)** Von Mises stress distribution of the fracture fragment for the 60% fracture height model. **(E)** Von Mises stress distribution of K-wires for the 15% fracture height model. **(F)** Von Mises stress distribution of K-wires for the 30% fracture height model. **(G)** Von Mises stress distribution of K-wires for the 45% fracture height model. **(H)** Von Mises stress distribution of K-wires for the 60% fracture height model.

In the horizontal displacement simulation (Figure 3), the 15% fracture height model showed a maximum fracture fragment displacement of 0.47 mm. The maximum stress in the fracture fragment was 73.95 MPa, while the peak stress on the K-wires reached 110.85 MPa. In the 30% fracture height model, the maximum displacement of the fracture fragment was 0.16 mm. The maximum stress in the fracture fragment was 77.25 MPa, while the peak stress on the K-wires reached 148.65 MPa. In the 45% fracture height model, the maximum displacement of the fracture fragment was 0.35 mm, with a maximum stress of 65.21 MPa in the fracture fragment and a peak stress of 223.73 MPa on the K-wires. In the 60% fracture height model, the fracture fragment displacement increased to 1.72 mm. The maximum stress in the fracture fragment was 73.95 MPa, while the peak stress on the K-wires reached 397.34 MPa, surpassing the yield strength of the material and resulting in substantial plastic deformation.

**FIGURE 4:**
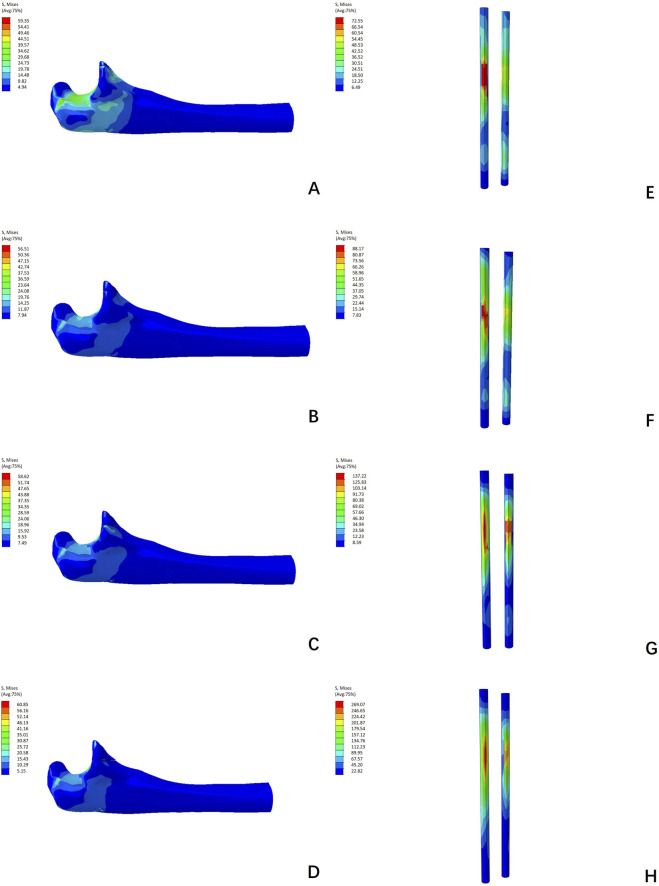
Von Mises stress distributions of the fracture fragments and K-wires in the rotational displacement simulation: **(A)** Von Mises stress distribution of the fracture fragment for the 15% fracture height model. **(B)** Von Mises stress distribution of the fracture fragment for the 30% fracture height model. **(C)** Von Mises stress distribution of the fracture fragment for the 45% fracture height model. **(D)** Von Mises stress distribution of the fracture fragment for the 60% fracture height model. **(E)** Von Mises stress distribution of K-wires for the 15% fracture height model. **(F)** Von Mises stress distribution of K-wires for the 30% fracture height model. **(G)** Von Mises stress distribution of K-wires for the 45% fracture height model. **(H)** Von Mises stress distribution of K-wires for the 60% fracture height model.

In the rotational displacement simulation (Figure 4), the 15% fracture height model showed a maximum fracture fragment displacement of 0.12 mm. The maximum stress in the fracture fragment was 59.35 MPa, with a peak stress of 72.55 MPa on the K-wires. In the 30% fracture height model, the maximum displacement of the fracture fragment was 0.11 mm. The maximum stress in the fracture fragment was 56.51 MPa, while the peak stress on the K-wires reached 88.17 MPa. In the 45% fracture height model, the maximum displacement increased to 0.15 mm. The maximum stress in the fracture fragment was 58.62 MPa, while the peak stress on the K-wires reached 137.2 MPa. In the 60% fracture height model, the fracture fragment demonstrated the largest displacement, reaching 0.18 mm, The maximum stress in the fracture fragment was 60.85 MPa, while the peak stress on the K-wires increased to 269.07 MPa.

## 4 Discussion

Nearly elbow fracture–dislocations involving the coronoid process primarily include posterolateral rotatory instability (PLRI), varus posteromedial rotatory instability (VPMRI), posterior Monteggia injury, and trans-olecranon fracture–dislocations (Pipicelli and King, 2020). Among these injury patterns, the terrible triad of the elbow represents a typical PLRI occurring in the extended elbow position. The coronoid process is one of the most important osseous structures contributing to elbow joint stability and plays a critical role in preventing posterior elbow dislocation as well as resisting anteroposterior shear forces (Ohl and Siboni, 2021; Rhyou et al., 2021). Previous studies have demonstrated that the coronoid process not only acts as a bony buttress, but also serves as the attachment site for several key soft tissue structures, including the anterior joint capsule of the elbow, the brachialis muscle, and the anterior bundle of the ulnar collateral ligament. Among the various components that constitute this stabilizing system, the coronoid process has been regarded as the “cornerstone” of elbow stability (Giannicola et al., 2020). These findings suggest that elbow stability is not determined solely by the bony height of the coronoid process; rather, the location of soft tissue attachments represents the critical factor determining whether a seemingly “Small-sized fracture” may lead to substantial joint instability (Ablove et al., 2006).

The terrible triad of the elbow is commonly managed through a lateral approach, with sequential repair of the coronoid fracture, radial head fracture, and the lateral collateral ligament (LCL) complex (Al-Ani et al., 2022). Zha et al. described a lateral minimal approach lasso technique for coronoid repair, in which the coronoid fragment can be repaired using sutures or fixed with K-wires inserted from posterior to anterior. This technique offers several clinical advantages, including technical simplicity, minimal surgical trauma, suitability for small fracture fragments, and ease of hardware removal (Zha et al., 2021). Some studies have reported that two K-wires inserted from the posterior or lateral aspect of the ulna toward the coronoid process can be used to cross-fix the fracture fragment, thereby providing sufficient stability while minimizing additional damage to small coronoid fragments (Choi et al., 2022; Egenolf et al., 2024). Giannicola et al. reported favorable clinical outcomes using fine-threaded K-wires for fixation of coronoid fractures in cases of complex elbow instability (Giannicola et al., 2013). In recent years, modified Kirschner wire techniques, including bent or hook-shaped K-wires, have also been proposed for the fixation of small coronoid fragments, with satisfactory clinical results reported (Jo and Shin, 2023). Consequently, double K-wire fixation has gradually emerged as a feasible method for the treatment of small coronoid fractures, particularly in cases where the fracture fragment is too small to accommodate standard screws. Due to the complex anatomy of the terrible triad of the elbow, diminutive fracture fragments often lack the volume to accommodate standard cortical screws, while extensive anterior exposure may compromise the vascular supply of the fragment. Moreover, although lasso suture techniques can restore soft tissue tension, they may provide limited resistance to rotational displacement and may not always maintain anatomical reduction. In this context, posterior-to-anterior fixation using two parallel K-wires offers several advantages, including minimal invasiveness, effective fragment capture, and multi-planar stabilization, making it an important technique for the management of coronoid fractures of varying heights.

In the pull-out simulation, significant variations in the relative displacement of the fracture fragment were observed among the four models within the loading range of 0–300 N. Displacement of the ulnar coronoid fracture fragment greater than 1.00 mm is generally considered detrimental to osseous union, while displacement greater than 2.00 mm is generally regarded as a loss of effective fixation (Esworthy et al., 2021). The 15% fracture height model reached 2.00 mm at a load of 169 N. Similarly, the 60% fracture height model reached 1.25 mm at a load of 300 N. Moreover, under identical loading conditions, the displacement of the fracture fragment in the 60% fracture height model was significantly greater than that in the other models, indicating that fixation stability in this group was insufficient to effectively maintain fragment apposition. In contrast, the 30% and 45% fracture height models demonstrated relatively small changes in fracture fragment displacement. Even at the maximum applied load of 300 N, the displacement in these two models remained below 2.00 mm, suggesting that double K-wire fixation provided substantial resistance to pull-out displacement for fractures involving 30% and 45% of the coronoid height. With respect to the stress experienced by the K-wires, the von Mises stress distributions demonstrated slightly more concentrated stress distribution in the 30% and 45% fracture height models compared with the other groups, indicating stronger resistance to pull-out forces under equivalent loading conditions. In contrast, the 15% fracture height model exhibited the lowest stress levels, suggesting that the load applied to the fixation construct was relatively dispersed. The 60% fracture height model demonstrated higher and more scattered stress distribution, indicating that the fixation effect on the fracture fragment was nearly ineffective. Regarding the stress distribution within the coronoid fracture fragment, the stress values remained relatively stable across all four models within the tested loading range and were maintained below 100 MPa. Among them, the 30% fracture height model exhibited a more homogeneous stress distribution compared with the other groups, suggesting that the fracture fragment experienced a more uniform load transfer under this fixation configuration. This relatively balanced stress distribution may reduce the risk of secondary fragment displacement and contribute to improved fracture healing, thereby reducing the incidence of delayed union or nonunion.

In the horizontal displacement simulation, the fracture fragment displacement in three of the four models remained below 1.00 mm within the tested loading range, except for the 60% fracture height model, indicating that these three models were able to achieve satisfactory fixation stability. In contrast, in the 60% fracture height model, the maximum displacement within the loading range escalated to 1.72 mm, indicating a loss of effective fixation stability. The 30% fracture height model exhibited a smaller displacement than the 45% fracture height model, indicating greater resistance to horizontal displacement. Regarding the stress distribution within the K-wires, the von Mises stress distributions showed that the 45% fracture height model exhibited relatively concentrated stress distribution. The maximum stress in the 60% fracture height model reached 397.34 MPa, which may approach the mechanical yield limit of the fixation construct and could potentially lead to irreversible deformation or even fracture of the K-wires. With respect to the coronoid fracture fragment, the stress values remained relatively stable in all four models within the tested loading range. Among them, the 45% fracture height model exhibited a more favorable stress distribution, suggesting that the fracture fragment experienced more uniform load transfer under this fixation configuration. Such a balanced stress distribution may reduce the risk of secondary fragment displacement and consequently decrease the likelihood of delayed union or nonunion.

In the rotational displacement simulation, the displacement of the fracture fragment in all four models remained below 1.00 mm, indicating that K-wire fixation provided adequate stability for coronoid fracture fragments of different heights under rotational loading conditions. Under identical loading conditions, the 30% fracture height model exhibited smaller displacement, suggesting that fixation stability of the fracture fragment was better maintained in this configuration. With respect to the stress experienced by the K-wires, the von Mises stress distributions showed that the stress on the fixation devices increased progressively with increasing fracture fragment height, demonstrating a trend toward stress concentration. This finding indicates that, under equivalent loading conditions, the 60% fracture height model experienced higher fixation stress, reflecting greater mechanical demand on the fixation construct during rotational loading. Regarding the stress distribution within the coronoid fracture fragment, the stress values remained relatively stable across all four models within the tested loading range and were consistently below 84 MPa. No obvious stress absorption phenomenon was observed at the fracture interface. These findings suggest that K-wire fixation can provide favorable biomechanical conditions for fracture healing under rotational loading conditions.

Among the four models, the 30% fracture height model demonstrated superior pull-out resistance, followed by the 45% fracture height model. In the horizontal displacement simulation, the 30% fracture height model demonstrated stronger resistance to horizontal displacement, whereas the 45% fracture height model exhibited more favorable stress distribution characteristics. In the rotational displacement simulation, fracture fragment displacement increased progressively with increasing fracture height; however, the magnitude of displacement remained within the micromotion range considered conducive to fracture healing. These findings suggest that K-wire fixation for coronoid fractures is particularly suitable for fractures involving approximately 30%–45% of the total coronoid height. Across the three loading conditions, the fracture height models showed relatively small differences in fracture fragment displacement in the horizontal displacement simulation. These findings indicate that double K-wire fixation provides a notable anti-rotational effect.

Results from the 15% fracture height model suggest that K-wire fixation is less effective for coronoid fragments smaller than approximately 2.7 mm. When integrated with the anatomical data from Ablove et al—who found that the mean distance from the anterior capsule insertion to the tip of the coronoid process is 2.36 ± 0.39 mm—this implies that nearly all Regan–Morrey type I coronoid fractures, even those that appear as very small avulsion injuries involving only the coronoid tip on imaging, will in fact be associated with complete or near-complete detachment of the anterior capsule once the fragment height exceeds 2.7 mm. Although the loss of bony support may appear minimal in such cases, the associated capsular disruption is substantial. If left untreated, during early postoperative rehabilitation, the absence of capsular restraint and tension frequently allows the elbow to re-subluxate in extension. While Pollock et al. demonstrated that Regan–Morrey type I anteromedial coronoid fractures with repair of the lateral collateral ligament (LCL) complex show stability comparable to that of a normal elbow (Pollock et al., 2009), such an approach that effectively ignores the coronoid fragment may still carry potential risks. Therefore, the authors believe that all coronoid fractures associated with the terrible triad of the elbow should, in principle, undergo anatomical reduction whenever possible, or at minimum capsular repair and fixation. Although the mean value proposed by Ablove provides an important reference, other studies have highlighted considerable inter-individual variation. For instance, one study measured this distance in 20 patients using MR arthrography and reported a mean of 1.7 ± 1.1 mm (Ablove et al., 2014), whereas the anatomical study by Shimura et al. found that the distance from the coronoid tip to the anterior capsular insertion averaged 5.8 mm, and emphasized the asymmetry of this attachment (Shimura et al., 2016). These morphological data highlight that such numerical references should not be applied rigidly in clinical practice. Instead, surgeons should evaluate the individual patient’s coronoid anatomy in conjunction with the 15% fracture height model, allowing for comprehensive assessment and individualized decision-making. Results from the 60% fracture height model suggest that K-wire fixation exhibits significant mechanical insufficiency under higher loading conditions when the coronoid fracture fragment exceeds approximately 10.8 mm in height. Previous anatomical studies have shown relatively consistent findings regarding the insertion of the brachialis muscle. Ablove et al. reported that the proximal insertion of the brachialis muscle is located at a mean distance of 10.13 ± 1.6 mm from the coronoid tip, while Weber et al. reported a distance of approximately 12.1 mm (Ablove et al., 2006; Weber et al., 2009). These findings suggest that when the fracture fragment height exceeds approximately 60% of the total coronoid height, the insertion of the brachialis muscle—one of the primary elbow flexors and an important dynamic stabilizer of the elbow joint—may be involved or affected. Under such circumstances, double Kirschner wire fixation generally serves only as a provisional stabilizer or supplementary fixation method, rather than as definitive fixation. In these cases, mini-plate fixation is typically required to provide sufficient structural support. This approach is essential to neutralize the substantial anterior shear forces generated by the humeral trochlea, thereby restoring physiological stability to the elbow joint and allowing for early mobilization.

The management of coronoid fractures associated with the terrible triad of the elbow has entered an era of precision-oriented treatment. The severity of coronoid fractures is determined not merely by the height of the fracture fragment, but more importantly by the extent of disruption to the soft-tissue anchoring structures attached to the coronoid process. Consequently, an evaluation and treatment strategy centered on joint stability rather than fragment height alone should be emphasized. Leveraging the double parallel K-wire fixation technique, which possesses distinct biomechanical advantages and minimally invasive characteristics, surgeons can achieve immediate stability and facilitate early functional mobilization. By integrating quantitative anatomical data with contemporary fixation strategies, this study proposes a comprehensive model for managing coronoid fractures of varying heights. Such an integrated strategy represents a robust pathway toward the precision treatment of complex elbow fracture–dislocations, effectively restoring joint stability and safeguarding against late functional impairment.

Several limitations of the present study warrant acknowledgment. First, the finite element models used in this study were simplified and represented idealized conditions. Potential concomitant injuries commonly associated with coronoid fractures, such as ligamentous and soft-tissue injuries or fractures involving other anatomical structures, were not incorporated into the models. Consequently, the calculated biomechanical outcomes may differ from those observed under actual clinical conditions. Therefore, the present findings should not be directly interpreted as evidence of overall elbow joint stability or as direct support for early postoperative mobilization in patients with the terrible triad of the elbow. Second, the materials in the finite element models were simplified as homogeneous isotropic materials. In addition, different K-wire diameters may exhibit different stress distributions and fixation characteristics. To isolate the effect of coronoid fracture height, the K-wire diameter was intentionally kept constant across all models. Therefore, the present study was designed to compare fixation performance among different fracture heights rather than to determine the optimal K-wire diameter. Further biomechanical investigations and prospective clinical studies are essential to validate these findings and assess their long-term efficacy. Future studies should incorporate multiple specimens with different ages, sexes, anatomical morphologies, and varying degrees of ligamentous instability to better replicate real-world injury conditions. Experimental validation is also warranted to further assess the robustness and clinical applicability of the current findings.

## 5 Conclusion

Within the established finite element models and loading conditions, dual parallel K-wire fixation provided effective stabilization when the fracture fragment height ranges from 30% to 45% of the total coronoid height, demonstrating relatively favorable biomechanical stability. Under these controlled conditions, this fixation strategy represent a relatively stable surgical option for the management of coronoid fractures associated with the terrible triad of the elbow and provide a biomechanical basis for postoperative rehabilitation and early mobilization. However, the simplified finite element models utilized in this study cannot fully replicate the complex injury mechanisms and instability patterns of the terrible triad of the elbow. Clinical decisions should be made in conjunction with the assessment of associated ligamentous injuries, radial head fractures, and overall elbow joint stability.

## Data Availability

The original contributions presented in the study are included in the article/supplementary material, further inquiries can be directed to the corresponding author.
